# CD200 expression in human cultured bone marrow mesenchymal stem cells is induced by pro‐osteogenic and pro‐inflammatory cues

**DOI:** 10.1111/jcmm.12752

**Published:** 2016-01-16

**Authors:** Charalampos Pontikoglou, Alain Langonné, Mamadou Aliou Ba, Audrey Varin, Philippe Rosset, Pierre Charbord, Luc Sensébé, Frédéric Deschaseaux

**Affiliations:** ^1^ EA3855 Université François Rabelais Tours France; ^2^ Department of Hematology University Hospital of Heraklion Heraklion Greece; ^3^ Etablissement Français du sang Centre‐Atlantique Tours France; ^4^ Stromalab Université de Toulouse UMR/CNRS 5273 U1031 Inserm EFS‐Pyrénées‐Méditerranée UPS Toulouse France; ^5^ Service d'orthopédie et traumatologie CHU Trousseau Chambray‐lès‐Tours France; ^6^ Inserm U972 and Université Paris XI Villejuif Cedex France

**Keywords:** mesenchymal stem/stromal cells, differentiation, osteoblasts, cell cultures, heterogeneity, NF‐κB

## Abstract

Similar to other adult tissue stem/progenitor cells, bone marrow mesenchymal stem/stromal cells (BM MSCs) exhibit heterogeneity at the phenotypic level and in terms of proliferation and differentiation potential. In this study such a heterogeneity was reflected by the CD200 protein. We thus characterized CD200^pos^ cells sorted from whole BM MSC cultures and we investigated the molecular mechanisms regulating CD200 expression. After sorting, measurement of lineage markers showed that the osteoblastic genes *RUNX2* and *DLX5* were up‐regulated in CD200^pos^ cells compared to CD200^neg^ fraction. At the functional level, CD200^pos^ cells were prone to mineralize the extra‐cellular matrix *in vitro* after sole addition of phosphates. In addition, osteogenic cues generated by bone morphogenetic protein 4 (BMP4) or BMP7 strongly induced CD200 expression. These data suggest that CD200 expression is related to commitment/differentiation towards the osteoblastic lineage. Immunohistochemistry of trephine bone marrow biopsies further corroborates the osteoblastic fate of CD200^pos^ cells. However, when dexamethasone was used to direct osteogenic differentiation *in vitro*, CD200 was consistently down‐regulated. As dexamethasone has anti‐inflammatory properties, we assessed the effects of different immunological stimuli on CD200 expression. The pro‐inflammatory cytokines interleukin‐1β and tumour necrosis factor‐α increased CD200 membrane expression but down‐regulated osteoblastic gene expression suggesting an additional regulatory pathway of CD200 expression. Surprisingly, whatever the context, *i.e*. pro‐inflammatory or pro‐osteogenic, CD200 expression was down‐regulated when nuclear‐factor (NF)‐κB was inhibited by chemical or adenoviral agents. In conclusion, CD200 expression by cultured BM MSCs can be induced by both osteogenic and pro‐inflammatory cytokines through the same pathway: NF‐κB.

## Introduction

Mesenchymal stem cells (MSCs), also referred to as multipotent mesenchymal stromal cells, skeletal stem cells and colony‐forming units‐fibroblasts (CFU‐Fs), are multipotent clonogenic stem cells able to self‐renew, to generate osteoblasts, chondrocytes and adipocytes and to expand extensively *in vitro* (reviewed in Charbord 2010) 1. As observed with other types of adult tissue stem cells, bone marrow BM MSCs exhibit heterogeneity at the phenotypic level and in terms of proliferation and differentiation potential. Indeed, expanded CFU‐Fs vary in size and in differentiation potential. Moreover, cells deriving from the same CFU‐F exhibit different morphology, phenotype and differentiation potential 2, 3, 4, 5, 6, 7. Furthermore, a previous study of our group on a significant number of MSC clones from human BM has clearly demonstrated that cultured cells are primed to various mesenchymal lineages and heterogeneity is obvious when comparing expression of the different differentiation markers from one clone to another 8. These data underline the complexity of the compartment of bone marrow cells which contains bona fide stem cells, but also progenitors, without yet clear understanding of the structure (hierarchical or stochastic) of the system. Moreover, the molecular mechanisms governing such heterogeneity have yet to be elucidated. Delineation of such mechanisms is expected to have great impact in devising more appropriate cell therapy protocols for the isolation of individual cell populations or the up‐grading of *ex vivo* cell amplification 9, 10.

In a previous study, we described a large panel of membrane molecules expressed by cultured BM MSCs 11. Among those molecules, CD200 appeared to be a marker of both native and cultured MSCs. CD200 is an immunoglobulin‐like protein broadly expressed by thymocytes, activated T cells, B cells, dendritic cells and vascular endothelial cells 12. When CD200 binds its specific receptor (CD200R), it induces an inhibitory signal 13. This generates immunosuppressive and tolerogenic cells, which are deleterious in a tumour context 14.

In this article, we studied how CD200 accounts for the heterogeneity in the MSC compartment, and we investigated the molecular cues that may be implicated in the expression of this molecule. We have found that expanded BM MSCs include different subpopulations of cells according to the membrane expression of CD200. On the one hand, in steady‐state conditions, CD200 high expression was found in cells with more pronounced osteoblastic features and it was easily up‐regulated to differentiate in the presence of pro‐osteogenic factors. On the other hand, in pro‐inflammatory context CD200 expression increased, whereas osteoblastic marker expression was down‐regulated. Interestingly, whatever the context (pro‐osteogenic or pro‐inflammatory) CD200 expression was under the control of the nuclear‐factor (NF)‐κB complex. CD200 appears therefore to be a molecule whose highly heterogeneous pattern of expression in MSC cultures is under the control of NF‐κB signalling pathway.

## Materials and methods

### Ethics statement

Bone marrow was aspirated from the posterior iliac crest of adults undergoing orthopaedic surgery after approval of the Medical Ethics Committee of Tours (named ‘Comité de Protection des Personnes Tours – CPP Région Centre [Ouest‐1]') and in accordance with their guidelines. Patients gave their written informed consent for the use of samples.

### Isolation and culture of BM MSCs

Mesenchymal stem cells were isolated as previously described 15. Briefly, BM nucleated cells were plated at 50,000 cells/cm^2^ in proliferation medium consisting in alpha‐modified Eagle's medium (αMEM; Invitrogen, Saint Aubin, France) supplemented with 2 mM l‐glutamine (Invitrogen), 100 U/ml Penicillin–Streptomycin (Invitrogen), 0.25 mg/l amphotericin B (Fungizone^®^; Bristol‐Myers Squibb, New York, NY, USA), 10% foetal calf serum (FCS; Logan, UT, USA) and 1 ng/ml fibroblast growth factor‐2 (R&D Systems, Inc., Minneapolis, MN, USA) and incubated in a humidified atmosphere with 5% CO_2_ at 37°C. The medium was changed twice a week. When cultures reached 70–90% confluency, cells were detached with trypsin/ethylenediaminetetraacetic acid (EDTA; Invitrogen) and replated at 1000 cells/cm^2^ (passage 1, P1). For the present study, all cells were derived from P2 cultures.

### Reagents

Human interleukin‐1‐beta (IL‐1β), tumour necrosis factor‐alpha (ΤΝF‐α) and human IL‐8 were purchased from R&D Systems, Inc. Human interferon‐gamma (IFN‐γ) was purchased from PeproTech (Rocky Hill, NJ, USA). The NF‐κΒ inhibitor pyrrolidinethiocarbamate ammonium (PDTC) was purchased from TOCRIS Biosciences (Bristol, UK). Dexamethasone (DXM) was purchased from Sigma‐Aldrich (St Louis, MO, USA); bone morphogenetic protein 2 (BMP2), BMP4 and BMP7 were purchased from R&D Systems, Inc.

### Cytokine induction

Mesenchymal stem cells were plated in six‐well plates at a concentration of 100,000 cells per well. Cells were then cultured for 3 days in the absence or presence of 20 ng/ml IL‐1β, 50 ng/ml ΤΝFα, 100, 200, 500 IU IFN‐γ, 10 ng/ml IL‐8, 10^−7^ M DXM, 50 ng/ml BMP2, 50 ng/ml BMP4, 50 ng/ml BMP7. In a separate set of experiments, MSCs were cultured for 7 days in the presence or absence of 50 ng/ml BMP2, 50 ng/ml BMP4, 50 ng/ml BMP7 with or without 10 mM PDTC.

### RNA extraction, PCR

Total RNA was extracted using TRIZOL reagent (Invitrogen) according to the manufacturer's instructions and cDNA was synthesized from 1 μg total RNA using PrimeScript^™^ 1st strand cDNA Synthesis Kit (Invitrogen). Real‐time quantitative PCR (QRT‐PCR),using gene‐specific primers (Table 1) was carried out on Bio‐Rad CFX96^™^ detection system (Bio‐Rad, Marnes‐la‐Coquette, France) using SsoFast^™^ EvaGreen^®^ Supermix (Bio‐Rad), according to the manufacture's recommendations. Twenty‐five nanogram cDNA were amplified as follows: 98°C for 3 min. following by 40 cycles at 98°C (2 sec.), 60°C (10 sec.) and 72°C (30 sec.). For all QRT‐PCR experiments *GAPDH* was used as a housekeeping gene and results are given by 2^−ΔCt^ or 2^−ΔΔCt^ method depending on the experiment. For classical RT‐PCR, SuperScript One‐Step RT‐PCR with Platinum Taq DNA polymerase (Invitrogen) was used, according to the manufacturer's instructions. The list of primers used is shown in Table 1.

**Table 1:** Primers used for (A) quantitative RT‐PCR, (B) classical RT‐PCR

Primer	Sequence
(A)	
GAPDH – *Forward*	5′‐CTGGCGCTGAGTACGTCG‐3′
GAPDH – *Reverse*	5′‐TTGACAAAGTGGTCGTTG A‐3′
CD200 – *Forward*	5′‐CCTAAGAATCAGGTGGGGAAGGA‐3′
CD200 – *Reverse*	5′‐GACGAGAAGAATTACCAGGGAAACA‐3′
DLX 5 – *Forward*	5′‐GCCACCAACCAGCCAGAGAA‐3′
DLX5 – *Reverse*	5′‐GACGAGAAGAATTACCAGGGAAACA‐3′
RUNX2 – *Forward*	5′‐GGCCCACAAATCTCAGATCGTT‐3′
RUNX2 – *Reverse*	5′‐CACTGGCGCTGCAACAAGAC‐3′
SOX9 – *Forward*	5′‐CAAGACGCTGGGCAAGCTCT‐3′
SOX9 – *Reverse*	5′‐TCTTCACCGACTTCCTCCGC‐3′
PPARG2 – *Forward*	5′‐AAGGCGAGGGCGATCTTGAC‐3′
PPARG2 – *Reverse*	5′‐GCAGGGGGGTGATGTGTTTG‐3′
ALPL – *Forward*	5′‐CCTGGAGCTTCAGAAGCTCAA‐3′
ALPL – *Reverse*	5′‐ACTGTG GAGACACCCATCCC‐3′
BGLAP – *Forward*	5′‐GAGGGCAGCGAGGTAGTGAAGA‐3′
BGLAP – *Reverse*	5′‐CGATGTGGTCAGCCAACTCG‐3′
(B)	
GAPDH – *Forward*	5′‐AATCCCATCACCATCTTCCAGG‐3′
GAPDH – *Reverse*	5′‐AGAGGCAGGGATGATGTTCTGG‐3′
CD200 – *Forward*	5′‐TGGTTTTCAGTTCCGCTATTGCT‐3′
CD200 – *Reverse*	5′‐ACCACATAACATGGCATTGCTTTAC‐3′
DLX – 5 – *Forward*	5′‐GCCACCAACCAGCCAGAGAA‐3′
DLX5 – *Reverse*	5′‐GACGAGAAGAATTACCAGGGAAACA‐3′
RUNX2 – *Forward*	5′‐AACTTCCTGTGCTCGGTGCTG‐3′
RUNX2 – *Reverse*	5′‐AGGGGTGTGTCATGTCCAGAGAGG‐3′
BGLAP – *Forward*	5′‐GTGCAGCCTTTGTGTCCAAGC‐3′
BGLAP – *Reverse*	5′‐GGGGAGGATTTGTGAAGACGG‐3′
ALPL – *Forward*	5′‐CTGGACCTCGTTGACACCTG‐3′
ALPL – *Reverse*	5′‐GACATTCTCTCGTTCACCGC‐3′
PPARγ2 – *Forward*	5′‐GGAGAAGCTGTTGGCGGAGA‐3′
PPARγ2 – *Reverse*	5′‐TCAAGGAGGCCAGCATTGTG‐3′

### Differentiation *in vitro*

To induce osteoblastic differentiation MSCs were plated at 1 × 10^4^ cells/cm^2^ in Lab‐Tek chamber slides (Nunc, Rochester, NY, USA). Upon confluence MSCs were cultured for 21 days in DMEM containing 4.5 g⁄l d‐glucose (Invitrogen) and supplemented with 10% FCS, 3 mΜ NaH_2_PO4 (Invitrogen) and 25 mg/l l‐ascorbic acid (Sigma‐Aldrich). Depending on the experiment 0.1 μM DXM (Sigma‐Aldrich) or 50 ng/ml BMP4 (R&D Systems, Inc.) were added. The osteogenic medium was changed every 2 days. To assess mineralization, cells were fixed with 4% formaldehyde and stained with 5% Alizarin red S (Sigma‐Aldrich) solution for 5 min. In addition, mineralized matrix was evaluated by von Kossa staining using 5% silver nitrate (Sigma‐Aldrich). Cells were subsequently stained with 5% sodium thiosulfate solution (Sigma‐Aldrich) for 5 min. To promote adipogenic differentiation, MSCs were plated at 1 × 10^4^cells/cm^2^ in Lab‐Tek chamber slides (Nunc). Upon confluence MSCs were cultured for 2 weeks in adipogenic medium consisting of low‐glucose DMEM supplemented with 10% FCS, 0.5 mΜ isobutyl‐methyl‐xanthine, 60 μΜ indomethacin and 10^−6^ M DXM (Sigma‐Aldrich). To reveal lipid droplets, cells were fixed with 4% formaldehyde and stained for 30 min. with Nile Red O (Sigma‐Aldrich). Nuclei were stained by 4,6‐diamidino‐2‐phenylindole (DAPI; AbCys, Paris, France).

### Flow cytometry cell sorting and analysis

For cell sorting, MSCs were labelled with unconjugated mouse anti‐human CD200 monoclonal antibody (mAb) or isotype control. Cells were subsequently incubated with a goat antimouse phycoerythrin (PE)‐conjugated secondary antibody (all purchased from BD Biosciences, San Jose, CA, USA). Cells were sorted using a MoFlo^™^ (Dako, Fort Collins, CO, USA) high speed cell sorter as previously described 11. Two fractions were thus isolated: CD200^pos^ and CD200^neg^ defined as the highest and lowest 30% of the CD200‐expressing cell populations respectively. For flow cytometry analysis, trypsinized MSCs were labelled with PE‐conjugated mouse anti‐human mAbs against, CD49a, CD49c, CD106 and CD146 (all purchased from BD Biosciences) or with an allophycocyanin‐conjugated CD200 mouse anti‐human mAb (eBioscience, San Diego, CA). In some experiments, cells were labelled with a mouse anti‐human PE‐conjugated alkaline phosphatase (R&D Systems, Inc.). The primary unconjugated anti‐human CD200 mAb (BD Biosiences) was also used in some experiments, along with an isotype‐matched mouse anti‐human IgG1 control mAb (BD Biosciences). Goat antimouse PE‐conjugated or a goat antimouse FITC‐conjugated secondary mAbs (BD Biosciences) were used. Flow cytometric analysis was performed with a BD FACSCalibur flow cytometer (BD Biosciences). Data were processed by means of CellquestPro^™^ software (BD Biosciences). Some sorted cells were also seeded into chamber slides for staining with anti‐human BGLAP (Thermo‐Fischer, Waltham, MA, USA) and anti‐CD200 antibodies.

### Immunohistochemical analyses

Human BM biopsy tissue was fixed in 4% paraformaldehyde, embedded in paraffin and sectioned at 6 μm. Sections were stained with haematoxylin and eosin (Sigma‐Aldrich). Immunostaining involved the primary antibody anti‐CD200 (R&D Systems, Inc.). Secondary antibodies were conjugated to HRP or to Alexa‐594. Some microsections were also co‐stained by a rabbit polyclonal anti‐human RUNX2 (Abcam, Cambridge, UK) recognized by secondary antibodies conjugate to Alexa‐488.

### Western blot analysis

The Western blotting was performed as previously described 15. Briefly, cultured CD200^pos^ and CD200^neg^ cells were detached with PBS/EDTA (10 mM) and lysed by lysis buffer (Sigma‐Aldrich). Protein concentration was determined using the Bradford Protein Assay (Bio‐Rad). Proteins were denatured by boiling for 3 min. in the presence of β‐mercaptoethanol and subsequently separated in SDS‐PAGE. Twenty micrograms of protein were loaded per lane. After separation, proteins were transferred onto polyvinylidene difluoride membranes which were then blocked by dried milk in TPBS buffer (0.1% Tween‐20/PBS) and incubated with anti‐αsmooth muscle actin (αSM‐actin, clone 1a4; Sigma‐Aldrich) or anti β‐actin (clone AC15; Sigma‐Aldrich) mAb overnight. After washing, membranes were incubated with peroxidase‐conjugated secondary antibodies (Bio‐Rad). Staining was revealed by chemiluminescence (ECLplus Western blotting detection kit from Amersham Biosciences, Saclay‐Orsay, France), and images were acquired with Chemi‐Smart 2000 using Chemocapt software (Vilber Lourmat, Marne‐la‐Vallée, France).

### Αdenoviral transduction of BM MSCs

Transduced BM MSCs were a generous gift of Dr RM Porter (Center for Advanced Orthopaedic Studies, Beth Israel Deaconess Medical Center, Harvard Medical School, Boston, MA, USA) and described previously 16. The adenoviral vectors encode the cDNA for a dominant negative super‐repressor ΙκΒ or for green fluorescent protein (GFP), both driven by the cytomegalovirus immediate early promoter.

### Statistical analysis

Grouped data are presented as mean ± S.D. of independent experiments. Data were analysed by means of non‐parametric Mann–Whitney test and by one‐way anova. The Wilcoxon matched‐pairs signed ranks test was used to compare experimental groups at steady‐state and after 3 days of treatment for CD200 expression.

## Results

### CD200 expression is heterogeneous

**Figure 1:**
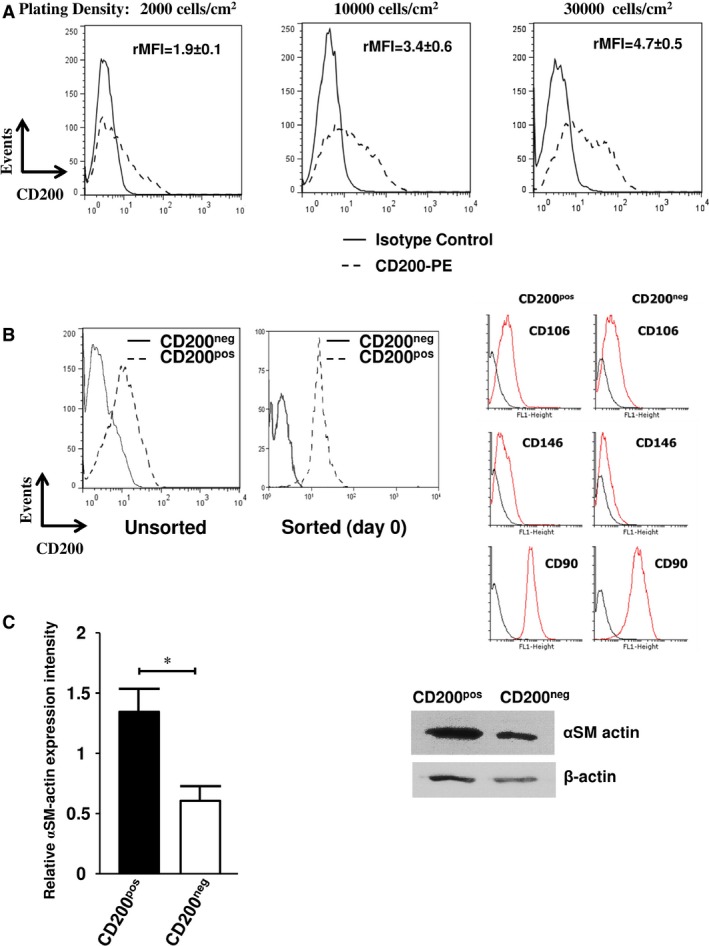
Expression of CD200 and αSM‐actin by cultured BM MSCs. (**A**) Flow cytometry analysis of cells from layers at different degree of confluence. Bone marrow MSCs seeded at 2000, 10,000 or 30,000 cells/cm^2^ were analysed after 2 days by flow cytometry following staining with PE‐conjugated control IgG (solid line) or PE‐conjugated CD200 antibody (dashed line). One representative experiment out of eight is depicted. The relative Mean Fluorescence Intensity (rMFI) was calculated and results are shown as mean ± S.D. (**B**) Cell sorting. Cultured BM MSCs were labelled with CD200 (left panel) and sorted to yield CD200^neg^ and CD200^pos^ subpopulations (middel panel). CD200pos and CD200neg cells were tested for CD146, CD106 and CD90 expressions (black line: negative control; red line: MSC marker tested). No significant differences were observed. (**C**) Expression of αSM‐actin in sorted cells. CD200^pos^‐ and CD200^neg^‐sorted subpopulations of MSCs were cultured for 10 days and tested for the expression of αSM‐actin. Left: αSM‐actin band intensity densitometry (normalization to β‐actin). Data expressed as mean relative band intensity ± S.D.; *P < 0.05. Right: Western Blots were performed by using anti‐αSM‐actin and anti‐β‐actin as loading control (representative experiment out of four).

The expression of CD200 protein by culture‐expanded MSCs was first assessed by flow cytometry. In all samples tested, CD200 protein was expressed by a subpopulation of MSCs. The percentage of CD200^pos^ cells (mean ± S.D.: 37.7% ± 13%, range 23–63.4%, *n* = 27) as well as the relative mean fluorescent intensity (rMFI) (3.7 ± 1.2, range 2.7–6.2) varied widely among donors. No apparent correlation could be established between the rMFI values or the proportion of CD200^pos^ cells and the age or sex of the donors (data not shown). CD200 expression significantly increased with cell density (*P* = 0.006 for the percentage of CD200^pos^ cells and *P* = 0.004 for rMFI) reaching maximal levels at 30 × 10^3^ cells/cm^2^ (Fig. 1A). Further increases in cell density had no effect on CD200 expression (data no shown).

**Figure 2:**
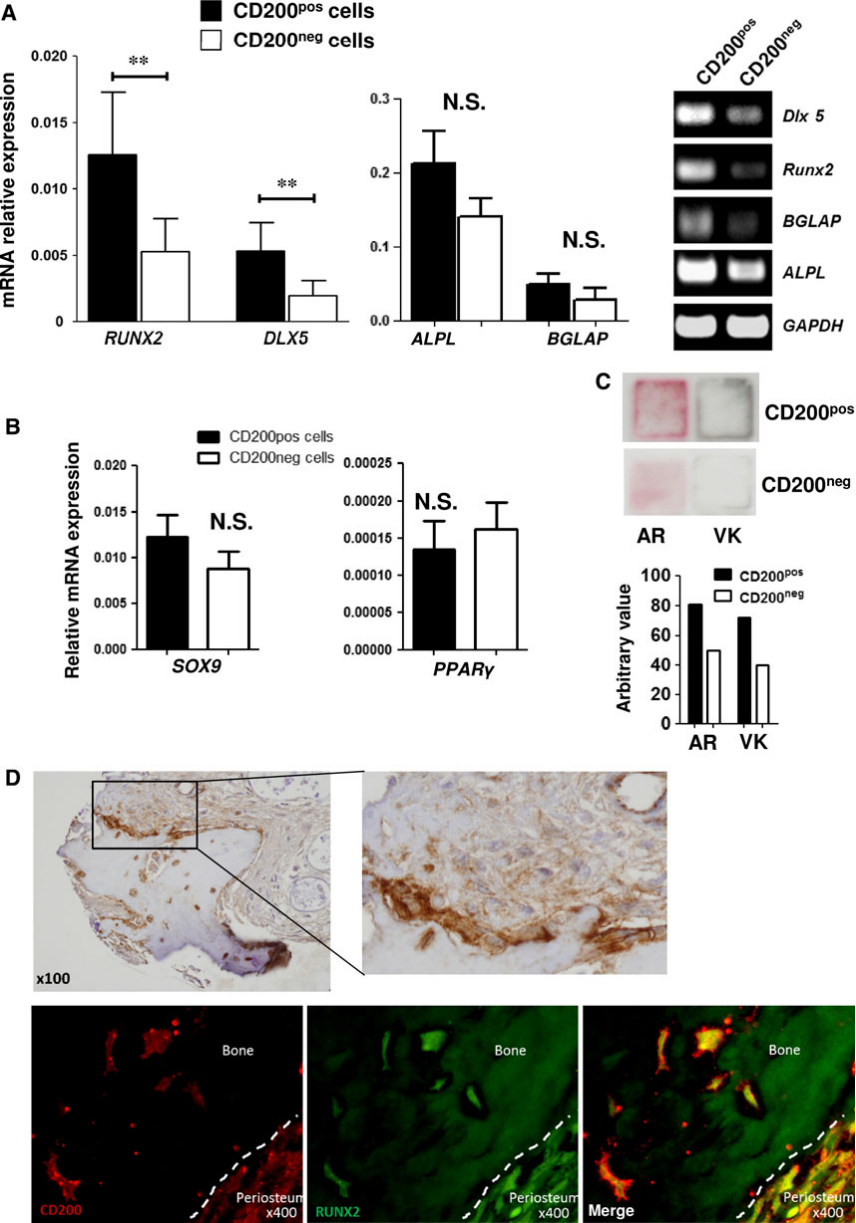
Analysis of lineage priming in sorted cells. (**A**) Osteoblastic lineage pathway. QRT‐PCR analysis (2^−ΔCt^ method using GAPDH as control) of *RUNX2*,*DLX5*, ALPL, BGLAP (left panel) mRNA levels in CD200^pos^ and CD200^neg^ cell fractions. Means ± S.D. of *n* = 4 experiments are shown **P < 0.01 Right panel: non‐quantitative RT‐PCR analysis of *RUNX2*,*DLX5*,*BGLAP ALPL* mRNA in CD200^pos^ and CD200^neg^ cell fractions. (**B**) Adipocyte and chondroblastic lineage pathways. QRT‐PCR analysis of PPARγ2 (adipogenic factor) and SOX9 (chondrogenic factor) mRNA levels in CD200^pos^ and CD200^neg^ subpopulations (2^−ΔCt^ method using GAPDH as control). Values are means ± S.D. of four experiments. N.S: Differences between means of CD200 subpopulations are not significant. (**C**) Mineralization of layers generated by CD200^pos^ and CD200^neg^ cells in the absence of osteo‐inducers. Alizarin Red (AR) and von Kossa (VK) staining of CD200^pos^ and CD200^neg^ cells cultured in medium containing 2%FCS/NaH_2_ PO_4_ and ascorbic acid. Bottom: Total staining density of each well was quantified using ImageJ software. (**D**) CD200 staining in BM microsections. Immunohistological (top) and immunofluorescence (bottom) detection of CD200^pos^ cells in human bone microsections. Top: Bone lining cells and cells embedded within bone matrix are CD200^pos^. A higher magnification of immune‐histochemical staining is shown. Bottom: CD200 (red)‐ and RUNX2 (green)‐positive cells are stained using anti‐human CD200 and anti‐human RUNX2 antibodies and recognized by fluorescent secondary antibodies.

We then investigated whether the different cell fractions (*i.e*. CD200^pos^ and CD200^neg^ cells) possessed different functional properties. Thus, the BM CD200^pos^ and CD200^neg^ MSC subpopulations were sorted by flow cytometry (Fig. 1B) and evaluated for the expression of molecules currently used to characterize MSCs. We were unable to detect differences in MSC membrane phenotype except for a higher, but not significant, proportion of CD106^pos^ and CD146^pos^ cells detected within the CD200^pos^ fraction, as compared to the CD200^neg^ one (Fig. 1B). The expression of αSM‐actin, a well‐known marker of cultured MSCs, was also assessed by WB in the CD200^pos^ and C200^neg^ subpopulations. Interestingly, the levels of αSM‐actin protein were significantly (*P* < 0.05) higher in CD200^pos^ cells (Fig. 1C). We then assessed the expression of the key adipogenic, chondrogenic and osteogenic transcription factors (*PPARγ2*;* SOX9*;* RUNX2* and *DLX5*, respectively) in CD200^pos^ and CD200^neg^ cells sorted from undifferentiated MSCs. Quantitative RT‐PCR analysis revealed a significant higher expression of *RUNX2* and *DLX5* in CD200^pos^ as compared to CD200^neg^ cells (*P* < 0.01 and *P* < 0.01, respectively) (Fig. 2A). Expressions of *PPARγ2* and *SOX9* were slightly reduced and increased respectively, but the differences were not significant when comparing the two subpopulations (Fig. 2B). To further demonstrate the intrinsically increased osteoblastic potential of CD200^pos^ cells, all fractions were placed in culture immediately following sorting. Cells were then cultured in phosphate‐containing medium without any osteo‐inducer to prevent any further commitment of all sorted fractions. On day 14 post‐sorting, a mineralized matrix deposition and sparse BGLAP expression could be detected in CD200^pos^ cells in contrast to CD200^neg^ cells (Fig. 2C and Fig. S1). This confirmed that CD200^pos^ fraction contained osteoblastic cells.

Moreover, we investigated CD200 expression directly in BM paraffin sections by using immunohistochemical methods. As shown in Figure 2D, non‐endothelial CD200^pos^ cells were localized along trabecular and cortical bone and were also found positive for RUNX2, confirming their osteoblastic phenotype. Interestingly, cells with extending cytoplasmic processes embedded in bone, *i.e*. osteocytes, stained also positive for CD200. Taken together, the increased osteoblastic gene expression along with the capacity to mineralize the extra‐cellular matrix and the *in situ* localization, indicate that CD200^pos^ population contains cells primed to the osteoblastic lineage and prone to easily differentiate into osteoblasts.

### CD200 expression is induced by pro‐osteogenic cues

**Figure 3:**
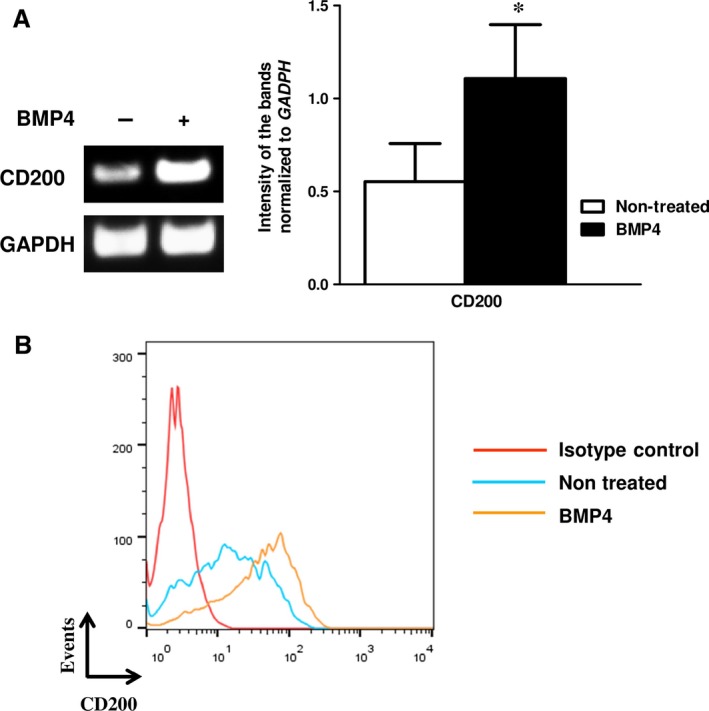
Effect of BMP4 on CD200 expression. MSCs were cultured for 7 days in the absence or presence of 50 ng/ml BMP4. (**A**) The effect of BMP4 on *CD200* mRNA expression was assessed by RT‐PCR. Left panel: a representative RT‐PCR analysis of CD200 mRNA in non‐treated or BMP4‐treated BM MSCs. Right panel: Band intensities from RT‐PCR results were quantified using ImageJ. Results were normalized to GAPDH. Means ± S.D. of *n* = 4 experiments are shown; *P = 0.04. (**B**) The effect of BMP4 on CD200 protein expression was assessed by flow cytometry following staining with PE‐conjugated control IgG or PE‐conjugated CD200 antibody (coloured lines; one representative experiment out of 3.

**Figure 4:**
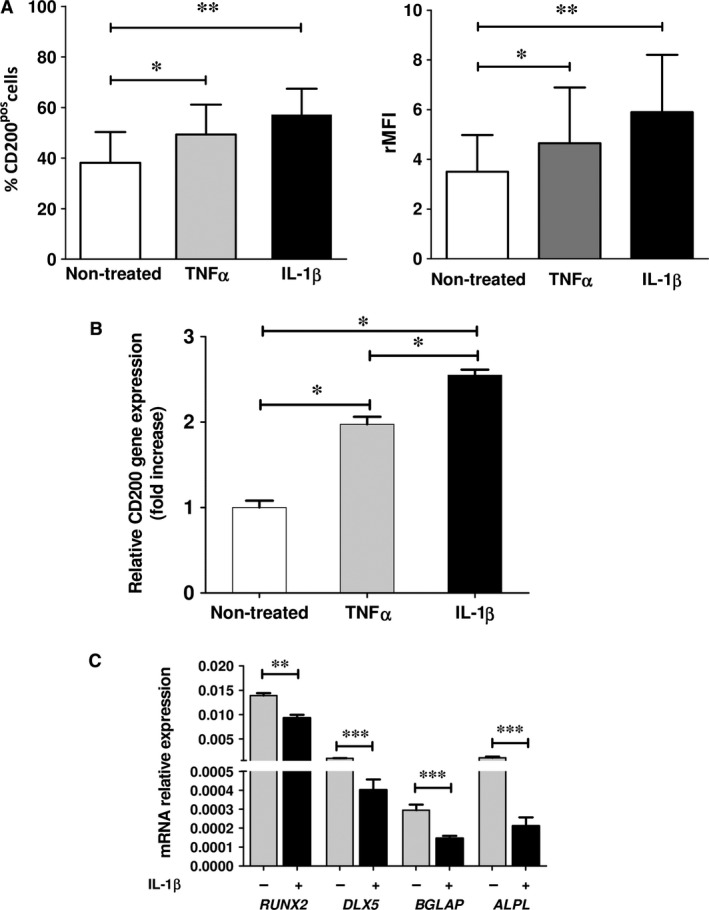
Effect of IL‐1β and TNF‐α on CD200 and osteoblastic‐related gene expression. (**A**) CD200 protein expression assessed by flow cytometry. Means of *n* = 8 experiments with percentage (left panel) and rMFI (right panel) of CD200^pos^ cells in cytokine treated or non‐treated BM MSCs; 3 days of treatment with 20 ng/ml IL‐1β or 50 ng/ml TNF‐α; *P < 0.05. (**B**) QRT‐PCR evaluation of *CD200* mRNA expression in non‐treated, IL‐1β‐ or TNF‐α‐treated MSCs. Results are reported as fold change relative to untreated MSCs and data represent means ± S.D. of *n* = 4 experiments; *P < 0.05. (**C**) BM MSCs were treated for 3 days with IL‐1β or TNF‐α as mentioned above or were left untreated and subsequently CD200^pos^ cells were sorted. *RUNX2*,*DLX5*,*BGLAP* and *ALPL* mRNA expression assessed by QRT‐PCR in sorted CD200^pos^ cell following treatment with or without IL‐1β (*n* = 4). Values correspond to 2^−ΔCt^ by using GAPDH as control; **P < 0.01; ***P < 0.001.

Because of the priming of CD200^pos^ cells to the osteoblastic lineage, we supposed that CD200 expression could be regulated by osteogenesis‐modulating factors secreted by MSCs. We therefore cultured cells for 3 days in the absence or presence of several osteogenic factors and assessed CD200 expression. Bone morphogenetic proteins 2, 4 and 7 were found to increase the expression of CD200 (increase at mRNA and protein levels) (Fig. 3). Interestingly, the effect of BMPs on CD200 protein expression became statistically significant when MSCs were treated for 7 days with the respective molecules (Table 2). Figure 3B displays a representative flow cytometry analysis of the effect of BMP4 on CD200 expression by BM MSCs and Figure 3A depicts the impact of BMP4 on CD200 gene expression as assessed by RT‐PCR. Notably, the same batch of cells has been used for Figure 4A and B left panel. qRT‐PCR analysis corroborated the positive effect of BMP4 on CD200 expression (data not shown). Moreover, whereas half of non‐treated (steady‐state) CD200^pos^ cells were also tissue non‐specific alkaline phosphatase (ALPL)‐positive (53.84 ± 12.86%), after BMP4 induction CD200 significantly increased (*P* = 0.0048) and nearly all CD200^pos^ cells co‐expressed ALPL (84.5 ± 9.84%) confirming their osteoblastic state (Table 3). On the other hand and in contrast to CD200^pos^ cells, BMP4 treatment had no effect in the proportion of ALPL^pos^ cells within the CD200^neg^ cell fraction (34.3 ± 12.23% and 37.93 ± 16.24% respectively; *P* = 0.376).

**Table 2:** Impact of BMP2,4,7 on CD200 expression by BM MSCs

	Non‐treated	BMP2	BMP4	BMP7
%CD200^+^	44.3 ± 18.6	67.3 ± 22.7	72.3 ± 21.4	66.8 ± 9.3
rMFI	3.2 ± 1.1	5.9 ± 1.5	7.6 ± 3.2	5.4 ± 1.5
*P*		0.014^a^	0.0045^a^	0.014^a^

a *P*‐value refers to difference in the percentage of CD200^+^ cells in treated *versus* untreated BM MSCs.

rMFI: relative mean fluorescence intensity; BM MSCs: bone marrow mesenchymal stem cells; BMP: bone morphogenetic protein.

**Table 3:** Impact of BMP4 on ALPL expression by CD200^+^ cells

	Non‐treated	BMP4
%CD200^+^	38.67 ± 9.39	67 ± 7.32
(%) ALPL expression within CD200^+^ fraction	53.4 ± 12.86	84.5 ± 9.84
*P*		0.0048^a^
*P*		0.0262^b^

a *P*‐value refers to difference in the percentage of CD200^+^ cells in non‐treated *versus* BMP4‐treated BM MSCs.

b *P*‐value refers to difference in the percentage of CD200^+^/ALPL^+^ cells within the CD200^+^ fraction cells in non‐treated *versus* BMP4‐treated BM MSCs.

BM MSCs: bone marrow mesenchymal stem cells; BMP: bone morphogenetic protein, ALPL: tissue non‐specific alkaline phosphatase.

### CD200 expression is also modulated in pro‐inflammatory contexts

In view of these findings, we further examined the effect of DXM, a factor widely used to direct *in vitro* osteogenic differentiation, on the expression of CD200 and ALPL by BM MSCs. As expected, 7‐day treatment with DXM resulted in an increase in the percentage of ALPL^+^ cells, as compared to the non‐treated group (71.36 ± 20.7% and 48.9 ± 11.46% respectively, *P* = 0.013; *n* = 8, Table 4). However, in striking contrast to BMP4, DXM treatment caused a significant decrease in the percentage of CD200^pos^ cells as compared to non‐stimulated cells (41.99 ± 9.67% and 26.02 ± 9.15% respectively, *P* = 0.04; *n* = 8, Table 4). Given the well‐established immune‐modulatory role of CD200 and the anti‐inflammatory function of DXM 16, we then focused on the potential effect of pro‐inflammatory molecules on CD200 expression (Table 5). IL‐8 and IFN‐γ had absolutely no effect on CD200 expression (*n* = 8; not shown). However, 3‐day treatment with IL‐1β or TNF‐α resulted in significant increase in CD200^pos^ cells (*P* < 0.002 and *P* < 0.01, respectively) as well as in up‐regulation of CD200 mRNA expression when compared to non‐treated cells (Fig. 4A and B, Fig. S2A and Table 5). Interestingly, in all experiments up‐regulation by TNF‐α was lower than that with IL‐1β.

**Table 4:** Impact of dexamethasone on ALPL and CD200 expression

	Non‐treated	DXM
%CD200^+^	41.99 ± 9.67	26.02 ± 9.15
(%) ALPL^+^	48.9 ± 11.46	71.36 ± 20.7
*P*		0.04^a^
*P*		0.0133^b^

a *P*‐value refers to difference in the percentage of CD200^+^ cells in non‐treated *versus* DXM‐treated BM MSCs.

b *P*‐value refers to difference in the percentage of ALPL^+^ cells in non‐treated *versus* DXM‐treated BM MSCs.

BM MSCs: bone marrow mesenchymal stem cells; DXM: dexamethasone, ALPL: tissue non‐specific alkaline phosphatase.

**Table 5:** Impact of IL‐1β, ΤΝF‐α and PDTC on CD200 expression by BM MSCs (*n* = 8)

	Non‐treated	TNF‐α	IL‐1β	PDTC	ΤΝF‐α+PDTC	IL‐1β+PDTC
%CD200^+^	38.1 ± 12.2	49.3 ± 11.8	57 ± 10	28.3 ± 7.7	41 ± 3.5	42.6 ± 5.1
rMFI	3.4 ± 1.4	4.6 ± 2.2	5.9 ± 2	2.5 ± 1	3.4 ± 0.7	3.5 ± 0.4
*P*		0.014^a^	0.0025^a^	0.0059^a^	0.042^b^	0.016^c^
*P* d					0.0885	0.0667

a *P*‐value refers to difference in the percentage of CD200^+^ cells in treated over non‐treated BM MSCs.

b *P*‐value refers to difference in the percentage of CD200^+^ cells in BM MSCs treated with TNF‐α+PDTC over cells treated with ΤΝF‐α only.

c *P*‐value refers to difference in the percentage of CD200^+^ cells in BM MSCs treated with IL‐1β+PDTC over cells treated with ΙL‐1β only.

d *P*‐value refers to difference in the percentage of CD200^+^ cells in BM MSCs treated with IL‐1β+PDTC or ΤΝF‐α + PDTC over non‐treated cells.

rMFI: relative mean fluorescence intensity; BM MSCs: bone marrow mesenchymal stem cells; PDTC: pyrrolidinethiocarbamate ammonium.

We next investigated whether induction of CD200^pos^ cells with IL‐1β or TNF‐α was also associated to overexpression of osteogenic markers. Following IL‐1β and TNF‐α treatment CD200^pos^ cells were sorted and evaluated for the expression of osteoblastic markers. Compared to unstimulated CD200^pos^ cells, IL‐1β‐stimulated CD200^pos^ cells expressed significantly lower levels of *RUNX2, DLX5*,* BGLAP* and *ALPL* (*P* < 0.01 *P* < 0.01, *P* < 0.01 and *P* < 0.01 respectively, *n* = 4) (Fig. 4C). A similar significant down‐regulation in osteoblastic gene expression was also detected in TNF‐α‐stimulated CD200^pos^ MSCs (not shown). Of note that IL‐1β was also able to modulate some osteoblastic markers (Fig. S2B).

**Figure 5:**
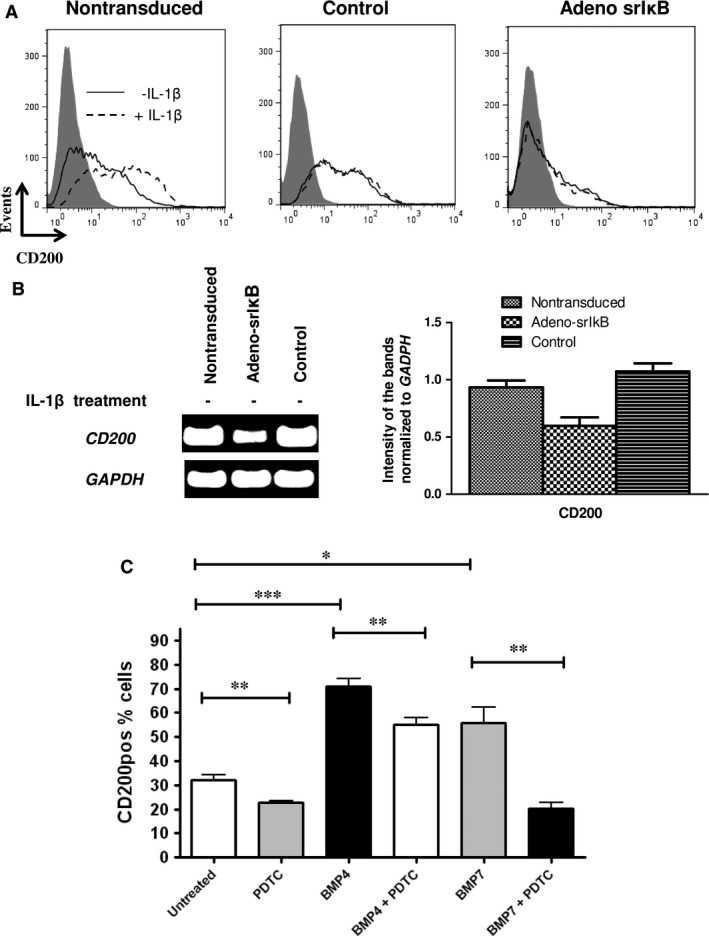
Effect of NFκB inhibition on CD200 expression. (**A**) Flow cytometry analysis of cells transduced with GFP‐ or srIκB‐encoding adenoviral vectors or untransduced. Grey‐filled histograms depict isotypic control; open continuous and dashed histograms depict CD200 expression by non‐treated and IL‐1β treated (20 ng/ml for 3 days) cells respectively. (**B**) RT‐PCR analysis of CD200 mRNA expression by non‐IL‐1β‐treated untransduced, adeno‐GFP (control) and adeno‐srIκΒ transduced cells. Left panel: One representative experiment out of three. Right panel: Band intensities from RT‐PCR results were quantified using ImageJ. Results were normalized to GAPDH. Means ± S.D. of *n* = 3 experiments are shown. (**C**) Effect of the NFκB inhibitor PDTC on BM MSCs. Cells were cultured for 7 days in the absence or presence of BMP4 (50 ng/ml), BMP7 (50 ng/ml) and DXM (10^−7^ M) with or without PDTC (10 mM). CD200 expression was assessed by flow cytometry. Cumulative results from *n* = 4 experiments are shown. *P < 0.05; **P < 0.01; ***P < 0.001.

As the ΝF‐κΒ pathway plays a key role in IL‐1β and ΤΝFα signal transduction 16, we investigated whether ΝF‐κΒ is implicated in the regulation of CD200 expression in cultured BM MSCs. Following stimulation with the respective cytokines, we found that the p65 subunit of the NF‐κB complex translocated to the nucleus of treated cells (Fig. S3A) in contrast to the absence of translocation in unstimulated cells. Interestingly, NF‐κB nuclear localization was more prominent following IL‐1β treatment (88.45 ± 12.65% cells) as compared to TNF‐α treatment (80.7%±15.34% cells), albeit not reaching statistical significance (*n* = 6 experiments). This finding could explain, at least in part the higher increase in CD200 expression following IL‐1β induction as compared to TNF‐α induction, as described above. In addition, flow cytometry analysis revealed that while in non‐treated cells, 30.4% ± 8.1% of CD200^pos^ cells co‐ expressed phosphorylated p65, following TNF‐α or IL‐1β induction percentages significantly increased (63.1% ± 13.2% and 70.2% ± 16.4%, respectively; *P* < 0.05 and *P* < 0.05, respectively; *n* = 6 experiments) (Fig. S3B). BM MSCs were then incubated with the chemical ΝF‐κΒ inhibitor PDTC in the presence or absence of ΙL‐1β or TNF‐α. As shown in Table 5, PDTC significantly decreased IL‐1β‐ or TNF‐α‐stimulated production of CD200 expression. Notably, the percentage CD200^pos^ cells following IL‐1β+PDTC or TNF‐α+PDTC treatment did not differ from that in non‐treated cells (Table 5). Furthermore, PDTC treatment resulted in a significant (*P* < 0.006) decrease in CD200 expression by MSCs, even in the absence of the pro‐inflammatory stimuli (Table 5). These data suggest that NF‐κB molecules play a key role in the regulation of CD200 expression. To probe more deeply into the role of NF‐κB pathway on CD200 expression, BM MSCs were transduced with an adenoviral construct encoding for a dominant negative inhibitor of ΝF‐κΒ (srIκΒ, a super‐repressor form of IκΒ) or a similar construct encoding GFP 16, 18 as control. Flow cytometry analysis revealed that the strong majority of cells were GFP^pos^, confirming the high efficacy of transduction (Fig. S4). Transduced or non‐transduced MSCs were cultured in the presence or absence of IL‐1β for 3 days and subsequently CD200 expression was evaluated. As shown in Figure 5A (right histogram) and Figure 5B, CD200 expression was indeed suppressed at both protein and mRNA levels in Adeno‐srIκB transduced cells, as compared to non‐transduced cells. Furthermore, IL‐1β treatment did not result in stimulation of CD200 expression in Adeno‐srIkB transduced cells (Fig. 5A middle histogram), whereas it increased CD200 expression in non‐transduced MSCs (Fig. 5A left histogram), as expected. In addition, transduction of MSCs with the GFP‐encoding adenoviral construct (control) resulted in an up‐regulation of CD200 expression at protein (Fig. 5A middle histogram) and mRNA levels (Fig. 5B), similar to those induced by IL‐1β treatment (Fig. 5A middle histogram). This finding may be attributed to the fact that the recombinant GFP‐encoding adenoviral vector used in our study has been shown to activate NF‐κΒ in MSCs to levels near those produced by IL‐1β 16, 18. This observation could also explain why in our study IL‐1β treatment did not further up‐regulate CD200 expression in adeno‐GFP‐transduced MSCs.

We finally investigated whether the CD200 up‐regulation because of osteogenic differentiation cues could also be abrogated by inhibiting NF‐κΒ signalling. Inhibition of NF‐κΒ function significantly (*P* < 0.001) diminished the effects of BMP4 and BMP7 on CD200 up‐regulation (Fig. 5C). The findings indicate that NFkB directly regulates CD200 expression downstream of both osteogenic as well as inflammatory cues.

## Discussion

In the present study, we show that the well‐established heterogeneity of culture‐expanded MSCs is also corroborated by the expression of the CD200 antigen. Although CD200^neg^ cells appear to show reduced osteoblastic lineage commitment as compared to CD200^pos^ cells, the latter population contains a contingent of cells primed to the osteoblastic lineage and prone to differentiate into osteoblasts. At the molecular level, the regulation of CD200 expression depends on NF‐κB pathway after induction of two types of stimuli: pro‐osteogenic and pro‐inflammatory ones.

The notion that CD200 expression is related to commitment/differentiation towards the osteoblastic lineage is supported by several arguments: (*i*) the increased expression of osteoblastic transcripts by CD200^pos^ cells, (*ii*) the induction of CD200 by BMPs, (*iii*) the propensity of CD200^pos^ cells to mineralize in the absence of osteogenic cues which fits well with their strong ALPL expression and (*iv*) the localization of CD200^pos^ cells along trabecular and cortical bone. Moreover, the up‐regulation of the vascular smooth muscle marker, αSM‐actin, in CD200^pos^ cells can be associated with the osteoblastic phenotype. Indeed, *in vivo* data show that αSM‐actin^pos^ cells line trabecular bone in both humans and mice and that Col2.3^pos^ osteoblasts express αSM‐actin in mice 19, 20, 21, 22. In addition, the increase in CD200 expression in confluent cultures is also consistent with osteoblastic commitment, as published data show that confluence enhances osteoblastic differentiation whatever the species (human, rodent, chicken) 23, 24, 25. We detected a non‐significant increase in *SOX9* in CD200^pos^ cells as compared to CD200^neg^ cells. SOX9 is a pro‐endochondral transcription factor and is currently found in osteochondroblastic progenitors 26, but SOX9 is also found in osteoblasts although at lesser extent than in chondroblasts 27. Thus, the slight increase in *SOX9* and the significant enhanced expression of *RUNX2*,* DLX5* and *BGLAP* factors are in agreement with the osteoblastic priming of CD200^pos^ cells. Finally, CD200 was recently reported as a marker of mouse MSC‐derived clones with osteogenic potential, whereas it was detected at very low levels in clones whose potential was restricted to adipocytes 25.

Our results show that CD200 expression by cultured human MSCs is regulated by the pro‐inflammatory cytokines TNF‐α and IL‐1β, which play critical roles in innate immunity. This is in agreement with the well‐known immune function of the CD200 molecule. Recent reports highlight the immune‐modulatory capacity of CD200^pos^ human BM MSCs by showing that these cells are able to suppress the secretion of TNF‐α by myeloid cells 28, 29 *via* the binding of CD200 expressed by MSCs to its receptor CD200R expressed by myeloid cells. Moreover, our experiments targeting the NF‐κB signalling pathway suggest that the regulation of CD200 expression in MSCs is mediated by the p65 subunit, a transcription factor of the canonical NF‐κB pathway. The regulation of CD200 expression by TNF‐α and IL‐1β in MSCs can be explained by the presence of NF‐κB binding site within the enhancer of CD200 distal promoter 30. Activation of NF‐κB appears not only to induce the expression of CD200 (our results) but also to prevent the osteoblastic differentiation 31, 32, 33. The negative regulation of the osteoblastic differentiation may be because of a direct effect of NF‐κB that inhibits *RUNX2* and *OSX* expression by binding to their promoter; alternatively the effect of NF‐κB may be indirect *via* activation of the JNK (c‐Jun N‐terminal kinase) pathway that up‐regulates *FOSL1* (also known as Fra1) expression, a transcription factor essential for bone formation after birth 32, 33. Our data show that CD200 expression depends on the NF‐κB pathway also in unstimulated MSCs. The precise mechanism of this effect has to be further elucidated. Non‐canonical NF‐κB signalling pathway and different forms of NF‐κB signalling transducers may be implicated 34. It is interesting to note that p100, a precursor of p52 (which interacts with p65 to induce pro‐inflammatory gene expression), can bind Alk2 (a BMP receptor). Although p52 decreases Alk2 function, p100 increases it, enhancing bone formation 35, 36. Therefore, irrespective of the type of activated NF‐κB pathway (canonical or non‐canonical), CD200 expression is induced, but the osteoblastic commitment could be either activated or inhibited according to the type of signalling.

As far as the functional role of CD200 within our experimental setting is concerned, we were not able to detect any effect following treatment with rhCD200 proteins or with inhibitory moAbs in cultured MSCs. Therefore, we have hypothesized that the function of CD200 may not be directed to MSCs or osteoblastic cells, but rather to haematopoietic cells. In favour of this hypothesis we have recently published an article suggesting 37 that CD200, expressed on the surface of MSCs, blocks osteoclasts' formation and their bone degradation capacity, thereby emerging as an important factor in the regulation of bone remodelling.

## Conflicts of interest

The authors confirm that there are no conflicts of interest.

## Supporting information


**Figure S1** Phenotypic analyses of CD200^pos^ and CD200^neg^ populations.

**Figure S2** Induction by pro‐inflammatory cytokines.

**Figure S3** Effect of IL‐1β and TNF‐α on canonical NF‐κB signalling in BM MSCs.

**Figure S4** Efficiency of gene transduction.
