# Supplementary figures and images for: CD200 expression in human cultured bone marrow mesenchymal stem cells is induced by pro‐osteogenic and pro‐inflammatory cues
Source: J Cell Mol Med. 2016 Jan 16;20(4):655–65. doi: 10.1111/jcmm.12752 (PMC5125749; doi:10.1111/jcmm.12752)

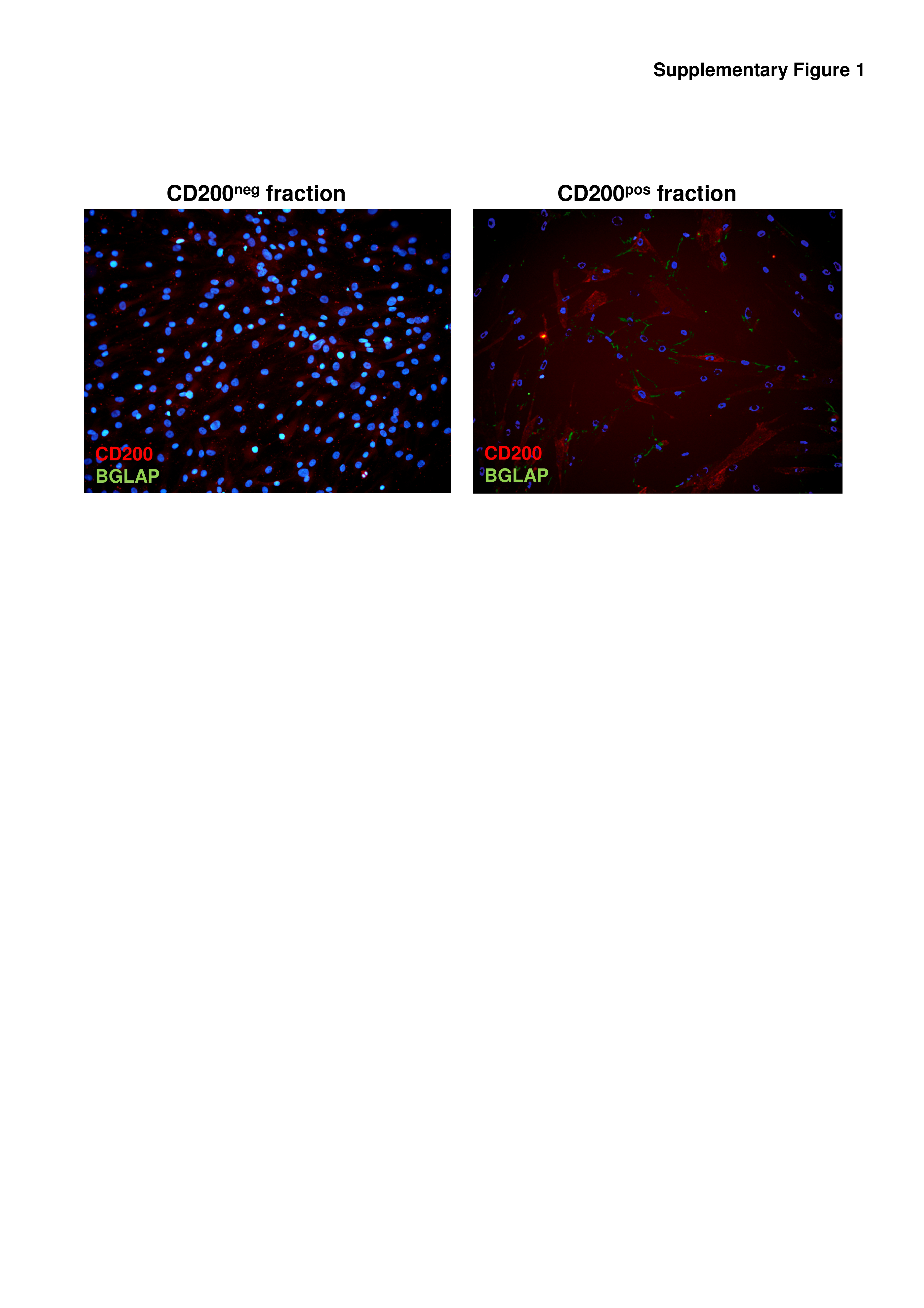

Supplement: Supplementary file 1 — Figure S1 Phenotypic analyses of CD200pos and CD200neg populations. [file JCMM-20-655-s001.tif]

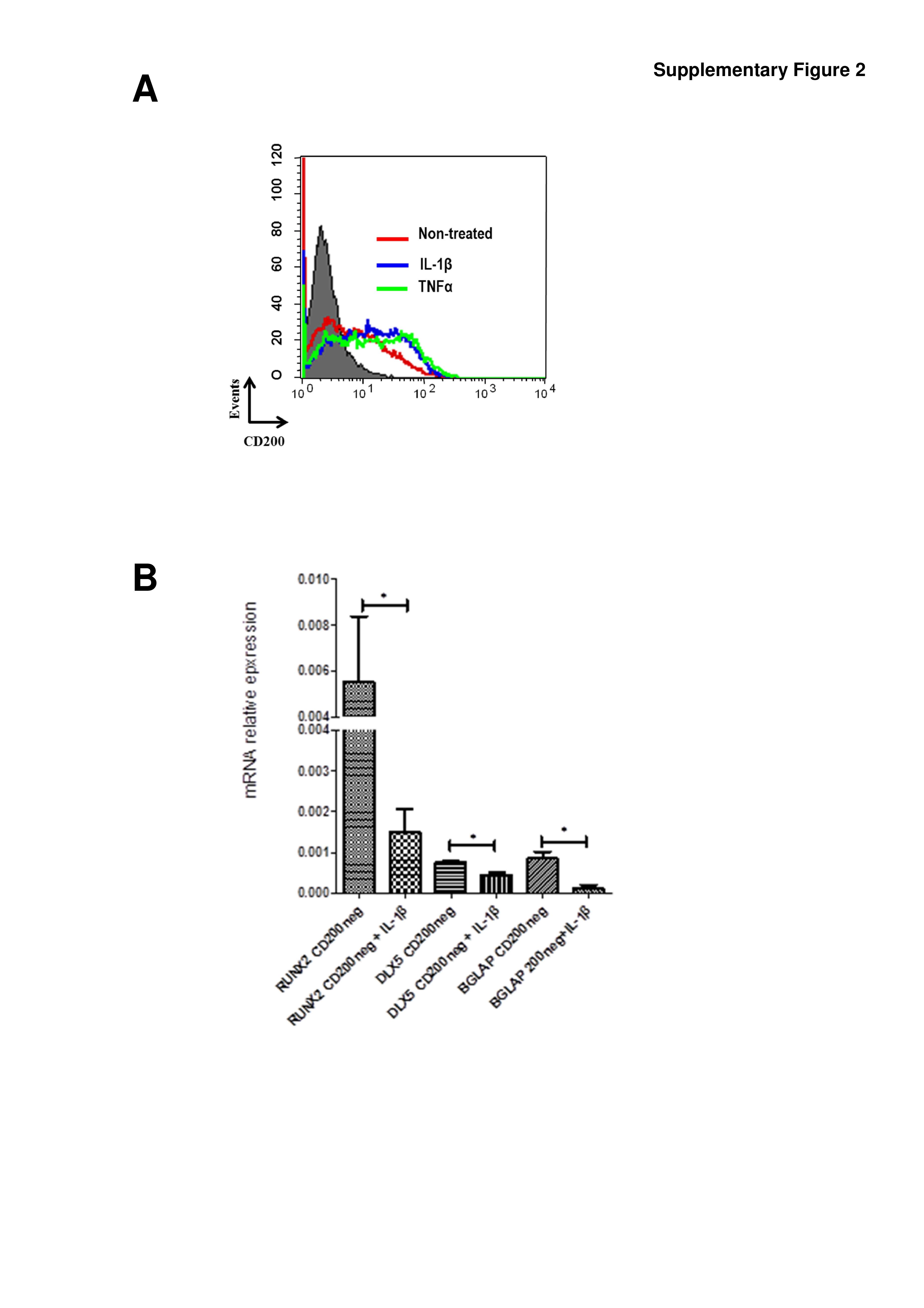

Supplement: Supplementary file 2 — Figure S2 Induction by pro‐inflammatory cytokines. [file JCMM-20-655-s002.tif]

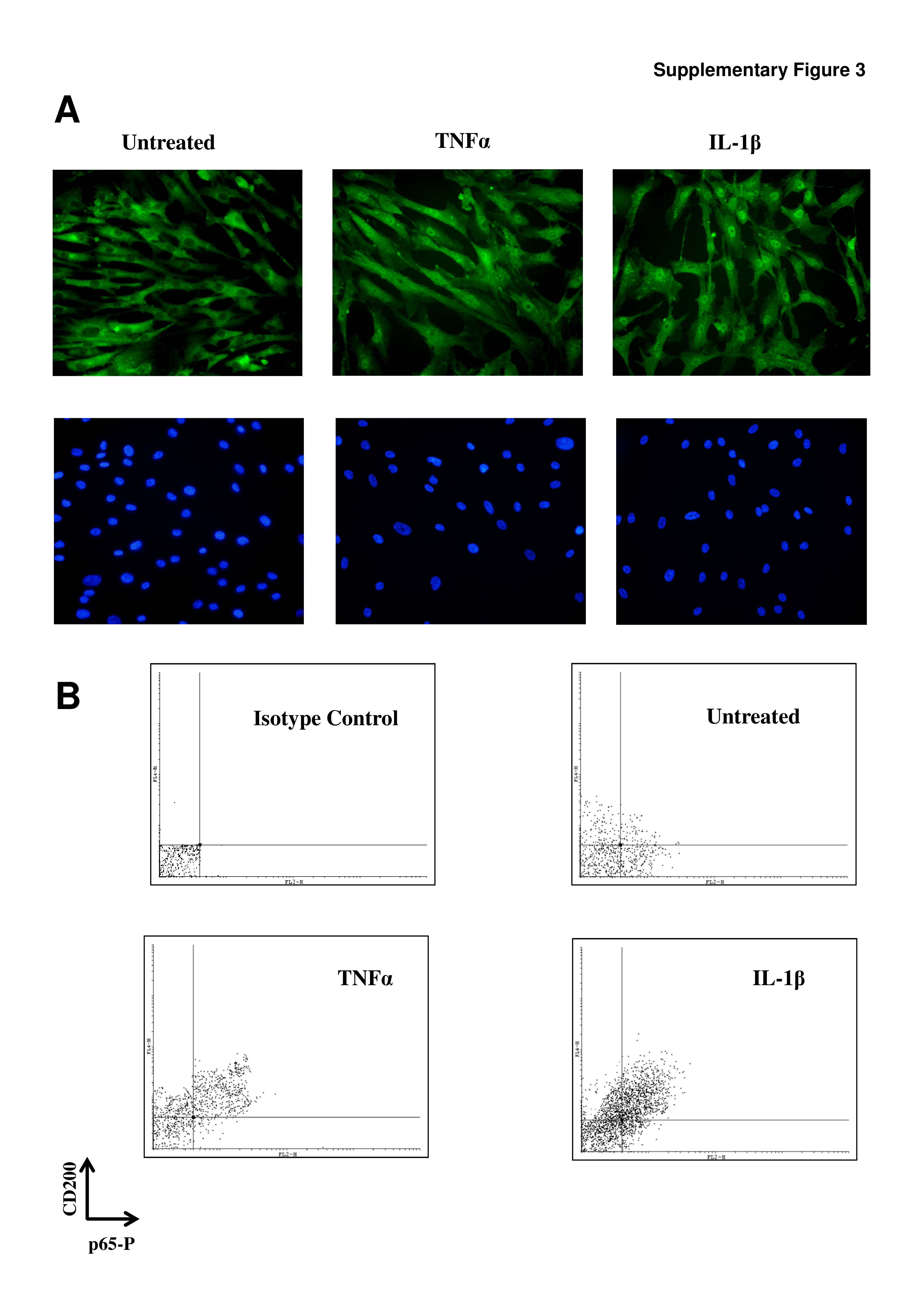

Supplement: Supplementary file 3 — Figure S3 Effect of IL‐1β and TNF‐α on canonical NF‐κB signalling in BM MSCs. [file JCMM-20-655-s003.tif]

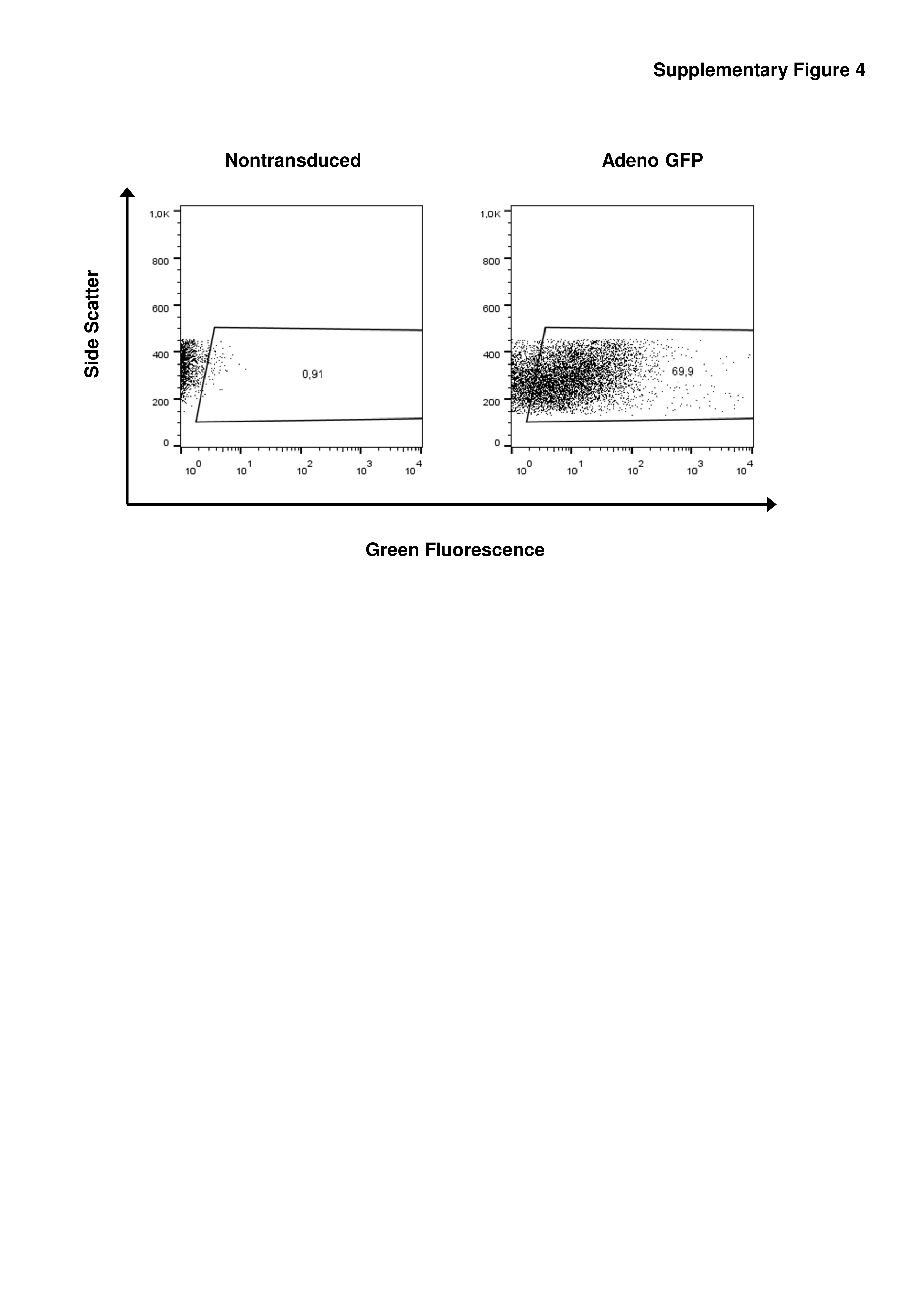

Supplement: Supplementary file 4 — Figure S4 Efficiency of gene transduction. [file JCMM-20-655-s004.tif]
